# Integrated breeding approaches to enhance the nutritional quality of food legumes

**DOI:** 10.3389/fpls.2022.984700

**Published:** 2022-09-07

**Authors:** Rintu Jha, Hemant Kumar Yadav, Rahul Raiya, Rajesh Kumar Singh, Uday Chand Jha, Lekshmy Sathee, Prashant Singh, Mahendar Thudi, Anshuman Singh, Sushil Kumar Chaturvedi, Shailesh Tripathi

**Affiliations:** ^1^Division of Genetics, ICAR-Indian Agricultural Research Institute, New Delhi, India; ^2^Department of Botany, Institute of Science, Banaras Hindu University, Varanasi, Uttar Pradesh, India; ^3^Crop Improvement Division, ICAR-Indian Institute of Pulses Research, Kanpur, Uttar Pradesh, India; ^4^Division of Plant Physiology, ICAR-Indian Agricultural Research Institute, New Delhi, India; ^5^Department of Agricultural Biotechnology and Molecular Biology, Dr. Rajendra Prasad Central Agricultural University, Samastipur, India; ^6^Shandong Academy of Agricultural Sciences, Jinan, China; ^7^Center for Crop Health, University of Southern Queensland, Toowmba, QLD, Australia; ^8^College of Agriculture, Rani Lakshmi Bai Central Agricultural University, Jhansi, Uttar Pradesh, India

**Keywords:** legumes, micronutrients, hidden hunger, anti-nutritional factors, biofortification

## Abstract

Global food security, both in terms of quantity and quality remains as a challenge with the increasing population. In parallel, micronutrient deficiency in the human diet leads to malnutrition and several health-related problems collectively known as “hidden hunger” more prominent in developing countries around the globe. Biofortification is a potential tool to fortify grain legumes with micronutrients to mitigate the food and nutritional security of the ever-increasing population. Anti-nutritional factors like phytates, raffinose (RFO’s), oxalates, tannin, etc. have adverse effects on human health upon consumption. Reduction of the anti-nutritional factors or preventing their accumulation offers opportunity for enhancing the intake of legumes in diet besides increasing the bioavailability of micronutrients. Integrated breeding methods are routinely being used to exploit the available genetic variability for micronutrients through modern “omic” technologies such as genomics, transcriptomics, ionomics, and metabolomics for developing biofortified grain legumes. Molecular mechanism of Fe/Zn uptake, phytate, and raffinose family oligosaccharides (RFOs) biosynthesis pathways have been elucidated. Transgenic, microRNAs and genome editing tools hold great promise for designing nutrient-dense and anti-nutrient-free grain legumes. In this review, we present the recent efforts toward manipulation of genes/QTLs regulating biofortification and Anti-nutrient accumulation in legumes using genetics-, genomics-, microRNA-, and genome editing-based approaches. We also discuss the success stories in legumes enrichment and recent advances in development of low Anti-nutrient lines. We hope that these emerging tools and techniques will expedite the efforts to develop micronutrient dense legume crop varieties devoid of Anti-nutritional factors that will serve to address the challenges like malnutrition and hidden hunger.

## Introduction

Malnutrition is one of the major challenging issues with the ever-increasing human population globally (Harding et al., 2018). It is widely prevalent in underprivileged population residing in the African and South Asian nations. The diets of almost one-third of the people across the globe is deficient in the required concentration of micronutrients in their food source (Stein, 2010). Malnutrition is gaining serious attention globally as it causes multiple health ailments due to insufficient daily intake of micronutrients including dietary minerals and vitamins in diet (Khoury et al., 2014). About 20 million infants are born annually with below average birth weight owing to micronutrient-related problems leading to neonatal death (Kumar and Pandey, 2020). High consumption of calorie-rich staple insufficient in micronutrition leads to “hidden hunger” especially in human population residing across low-income countries (Dawson et al., 2019). Among the various micronutrient deficiencies, iron deficiency is the most commonly found micronutrient deficiency causing anemia especially, in women and children human population globally (Abbaspour et al., 2014). About 2 billion people are afflicted with iron deficiency across the globe (Viteri, 1998; Miller, 2013). Zinc deficiency is associated with impaired growth and development, compromised immune response-related issues, cardiovascular disease, and chronic kidney disease in human population (Nakatani et al., 2021). Similarly, inadequate folate consumption affects millions of people and enhances women’s risk during the pregnancy (Sen Gupta et al., 2013). Grain legumes are well known for their high protein and vitamin content and are affordable alternatives to animal protein. The United Nations (UN) declared the year 2016 as “International Year of Pulses (IYOP, 2016)” to create awareness about pulse crops as vital components for global food and nutritional security and to promote pulse production. Food legumes such as chickpea (*Cicer arietinum* L.), common bean (*Phaseolus vulgaris L*.), lentil (*Lens culinaris*), mung bean (*Vigna radiata* L.), pea (*Pisum sativum*), and pigeon pea (*Cajanus cajan* [L.] Millsp.) are rich reservoir of dietary micronutrients and essential amino acids and thus could potentially play a pivotal role in overcoming the increasing challenges of malnutrition-related problems.

Understanding the process of mineral acquisition, transport, and accumulation in legume seeds is critical for optimizing nutrient bioavailability (Roorkiwal et al., 2021). Each of these processes is likely governed by a set of genes, many of which are still unknown. Several studies have discovered genes involved in uptake and mobilization of Fe (Palmer and Guerinot, 2009; Xiong et al., 2012; Sen Gupta et al., 2017) and Zn (Palmgren et al., 2008; Urwat et al., 2021) from different parts of plant to the seeds.

Improving grain micronutrient contents through plant breeding approach is an economically viable options to overcome the malnutrition problem. Crop biofortification requires a multidisciplinary effort involving the plant breeders, nutritionists, and agronomists. Thorough assessment of global legume germplasm including cultivated and wild species could greatly assist us in identifying high micronutrient containing genotypes. Pre-breeding and breeding efforts are needed to develop the micronutrient dense lines and test them at multilocations to assess the role of environment on micronutrient assimilation. Likewise, increasing legume genomic repertoire could enable us uncovering the various QTLs/genomic segments controlling micronutrient content through biparental QTL mapping and genome-wide association studies (GWAS). Likewise, unprecedented advancements in functional genomics shed light on the various underlying candidate gene(s) regulating various micronutrient biosynthesis and accumulation with precise function. Similarly, agronomic interventions like foliar application of fertilizers enriched with micronutrients could also aid in the biofortification of grain legumes are mentioned (Poblaciones and Rengel, 2016; Silva et al., 2019). Transgenics for improving micronutrient content with suitable examples are illustrated, and scope of genomic selection, speed breeding and emerging genome editing and microRNA technologies for designing biofortified grain legumes are discussed. An integrated breeding approach embracing all these technologies can surely help us in alleviating the rising malnutrition-related problems and securing “zero hunger” world. Additionally, despite the fact that grain legumes are a rich source of minerals, several Anti-nutritional elements or factors, including as phytic acids (phytate or PA), oxalates, and tannins, chelate the minerals and reduce their bioavailability by limiting their absorption in our dietary system, thus numerous health issues occur. Several studies demonstrated the presence of phytic acids (Urbano et al., 2000; Shi et al., 2018; Pramitha et al., 2021), RFOs (Zhawar et al., 2011; Roorkiwal et al., 2021), oxalates (Noonan and Savage, 1999; Shi et al., 2018) and tannins (Arts et al., 2000; Petroski and Minich, 2020) in legumes and their Anti-nutritional properties.

## Molecular mechanism involved in uptake of Fe and Zn in legumes

Metal micronutrients play critical biochemical and structural roles in plants. Terrestrial vascular plants, as sessile organisms, are exposed to a wide range of environmental circumstances and the stresses that come with them, including soil nutrient deficiency signals, which have a detrimental impact on growth and development (Fan et al., 2021). Understanding the process of mineral uptake from the soil and their accumulation in legume seeds is essential for nutrient bioavailability and optimization. Several studies have attempted to identify and establish gene families involved in mineral transport from different vegetative tissues of plant to seed. In recent years, much has been learned about the genes and proteins necessary for primary Fe and Zn uptake from the soil but here we attempt to illustrate the Fe and Zn transport mechanisms in legumes.

### Fe transport

**Figure 1:**
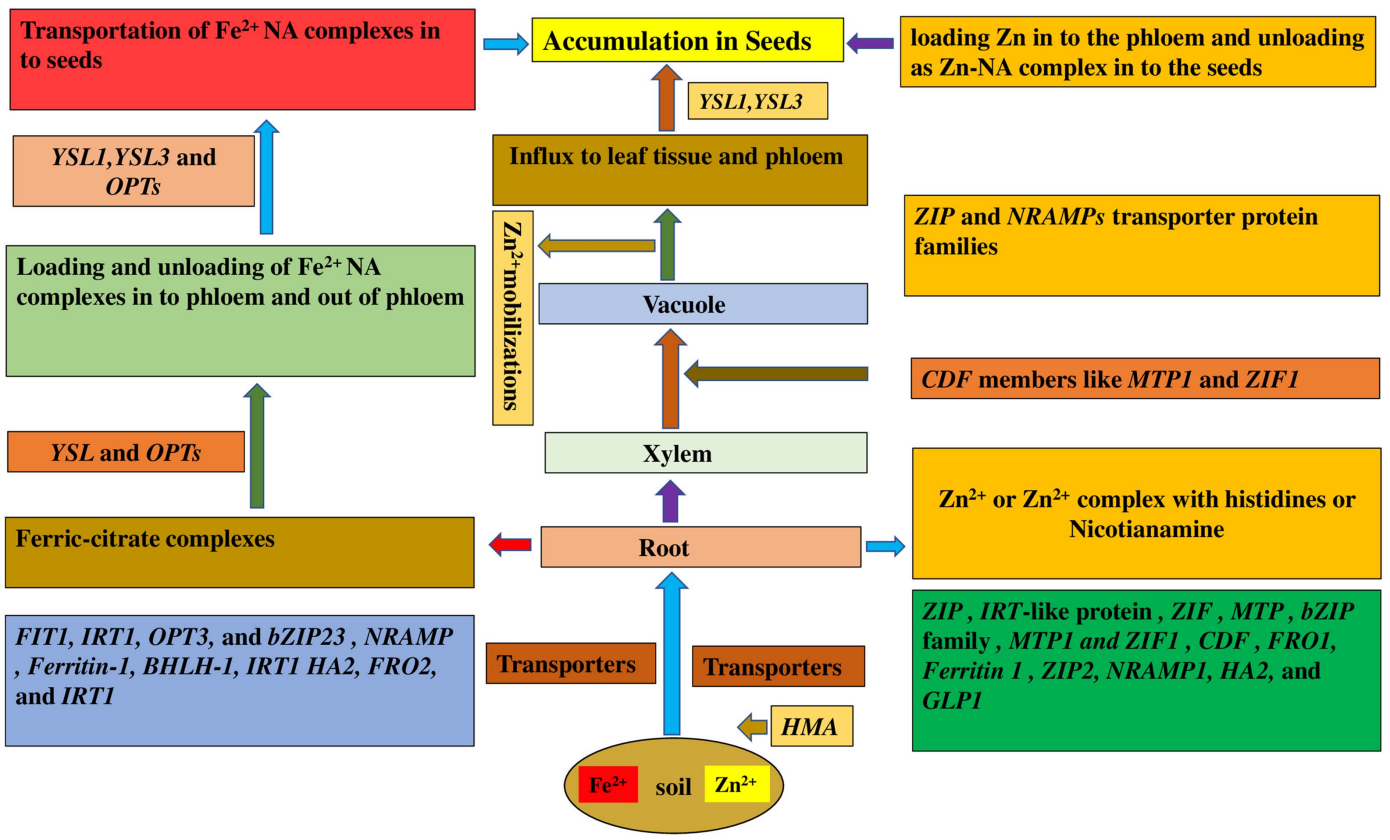
Molecular mechanism involved in uptake and acquisition of Fe and Zn in legume seeds. YSL, Yellow Stripe Like; OPT, Oligopeptide transporter; NRAMP, Natural resistance-associated macrophage protein; IRT, Iron regulated transporter; FER, Ferritin; FRO Ferric reduction oxidase; ZIP, Zinc induced protein; HA, H+ transporting ATPase; GLP, Germin like protein; CDF, Cation diffusion facilitator; MTP, Metal tolerance protein; ZIF, Zinc-induced facilitator; BHLH, basic helix–loop–helix; HMA, heavy metal ATPase.

In legumes, the proteins HA2 (H^+^-ATPase), FRO2 (Ferric reductase oxidase2), and IRT1 (iron-regulated transporter) are primarily responsible for Fe absorption and delivery to the roots (Figure 1; Roorkiwal et al., 2021). A plasma membrane-localized transporter protein, YS1, is a crucial protein for acquiring Fe *via* uptake of Fe(III)-phytosiderophores (Curie et al., 2001). Expression analysis has revealed several genes that encode Fe transporter protein families in several legume species such as in *Arachis hypogaea* (AhIRT1, Xiong et al., 2012), *Medicago truncatula-* natural resistance-associated macrophage protein 1 (MtNRAMP1, Tejada-Jiménez et al., 2015; MtZIP3, MtZIP5, and MtZIP6, Lopez-Millan et al., 2004), *Glycine max* (NRAMP, Qin et al., 2017)*, Lens culinaris* [Ferritin-1, basic helix–loop–helix-1 (bHLH-1), and FER-like transcription factor protein and IRT1, Sen Gupta et al., 2017] and in *Cicer arietinum* (CaFer1, Parveen et al., 2016). Fe sensing mechanisms are still unknown, despite the discovery of distinct transcriptional networks that regulate absorption and intracellular distribution (Thomine and Vert, 2013). The high-affinity transporter IRT1 is principally responsible for Fe absorption (Palmer and Guerinot, 2009). Transporters like ZIP (zinc inducible protein) and YSL (yellow stripe like) are implicated in metal unloading from the xylem (Roorkiwal et al., 2021). In *Arabidopsis*, the oligopeptide transporter (OPT3) has been proposed to play a key role in accurate long-distance Fe transmission from shoots to roots, as well as in importing Fe into phloem companion cells (Kumar et al., 2017). The NRAMP (NRAMP3 and NRAMP4) family of genes is known to play a role in Fe homeostasis, while YSL and OPTs are involved in the loading and unloading of Fe^2+^ NA (nicotianamine) complexes into and out of phloem (Palmer and Guerinot, 2009).

Mutants with defects in homeostatic regulation of Fe and other metals have been studied for several years. More recently, it has been shown that the *Ferric reductase defective3* (*FRD3*) protein controls Fe localization within the plant (Green and Rogers, 2004). It was observed in pea (*Pisum sativum*) mutant bronze (brz) that there is overaccumulation of Fe, Cu, Mn, Zn, and other elements in shoots (Cary et al., 1994), and the pea mutant degenerative leaves (dgl) overaccumulates Fe in leaves and seeds (Grusak et al., 1996). Given evidences suggest for the presence of nicotianamine (NA) in shoot tissues and its affinity for different ions. NA is a non-protein amino acid that is ubiquitous in plants. NA has been implicated as important for translocation of Cu, Fe, Mn, and Zn (Pich et al., 1994; Pich and Scholz, 1996). Based on studies of the *chln* mutant, NA is vital for homeostasis of Fe and other metal micronutrients. The tomato mutant *chloronerva* (*chln*) exhibits increased ferric reductase activity and overaccumulates Fe in mature leaves (Stephan and Grun, 1989), yet the younger leaves are chlorotic (Becker et al., 1992). In *Arabidopsis*, the combination of null mutations in two *AtYSL*s, *YSL1* and *YSL3*, resulted in a severe phenotype including interveinal chlorosis; altered metal concentrations in leaves, roots, and seeds; and greatly decreased fertility. YSL promoter-*β-glucuronidase* (*GUS*) reporter constructs reveal a localization pattern that was consistent with a role for YSL1 and YSL3 in providing metal-NA compounds to leaves, pollen, and developing seeds (Waters et al., 2006).

Metal remobilization from senescent leaves is a major role of *YSL* genes, which also serve as transporters in seed development, reproductive organ growth, and long-distance transport of metal complexes with NA (Di Donato et al., 2004). Iron and Cu regulate *AtYSL2* expression, and *YSL2* is abundantly expressed in the root endodermis, pericycle, and xylem cells. *AtYSL2* is thought to play a function in transporting metals to and from the vasculature based on its location. Downregulation of the genes *YSL1 and YSL3* in Fe-deficiency situations would decrease Fe removal from the xylem into adjacent tissues. Hence, the physiological role of YSL1 and YSL3 is to translocate metals into vascular parenchyma cells for distribution away from veins into interveinal regions in maturing leaves, away from interveinal regions toward phloem tissue in senescing leaves, and out of the vasculature in fruits to supply seeds.

### Zn transport

Zinc is primarily taken up as Zn^2+^ across the plasma membrane of root cells in legumes. Zn absorption and transport from root to seed have been aided by ZIP (Zinc Iron Permease) transporters (Palmgren et al., 2008). The key transport protein families involved in Zn transport are the ZIP family/ZRT, IRT-like proteins, the HMA (Heavy Metal ATPases), and the MTP (Metal Tolerance Protein) family (Gupta et al., 2016). ZIP genes have been identified in various plant species like *Arabidopsis* (bZIP19, bZIP23, and bZIP24, Assunçao et al., 2010), Soybean (*GmZIP1*; Moreau et al., 2002), *M*. *truncatula* (MtZIP1, MtZIP3, MtZIP4, MtZIP5, MtZIP6, and MtZIP7, Lopez-Millan et al., 2004) and in *Phaseolus vulgaris* (PvZIP12, PvZIP13, PvZIP16, and PvbZIP1, Astudillo et al., 2013).

Net Zn fluxes in the shoot are influenced by both symplastic and apoplastic fluxes. Apoplastic Zn transport includes Zn entering the cytosol *via* the cell wall plasma membrane contact, making it less selective than symplastic transport. Symplastic transport, on the other hand, regulates the selectivity and amount of nutrient delivery (Gupta et al., 2016). MTP1 and ZIF1 transporters, members of the cation diffusion facilitator (CDF) family, are involved in Zn sequestration in vacuole, while NRAMPs are involved in Zn mobilization from the vacuole (Haydon and Cobbett, 2007). Zn is loaded into the xylem by HMA (Heavy metal ATPases), and is transported as Zn^2+^ or in complex with histidines or Nicotianamine within the xylem (Palmgren et al., 2008). YSL is involved in loading Zn into the phloem and discharging it to the seeds as the Zn-NA complex, whereas members of the ZIP family are involved in mediating Zn^2+^ influx to leaf tissue and the phloem (Waters and Grusak, 2008). The molecular mechanism of uptake and mobilization of Zn^2+^ from root to seeds have been reviewed in several articles (Waters et al., 2006; Palmgren et al., 2008).

**Figure 2:**
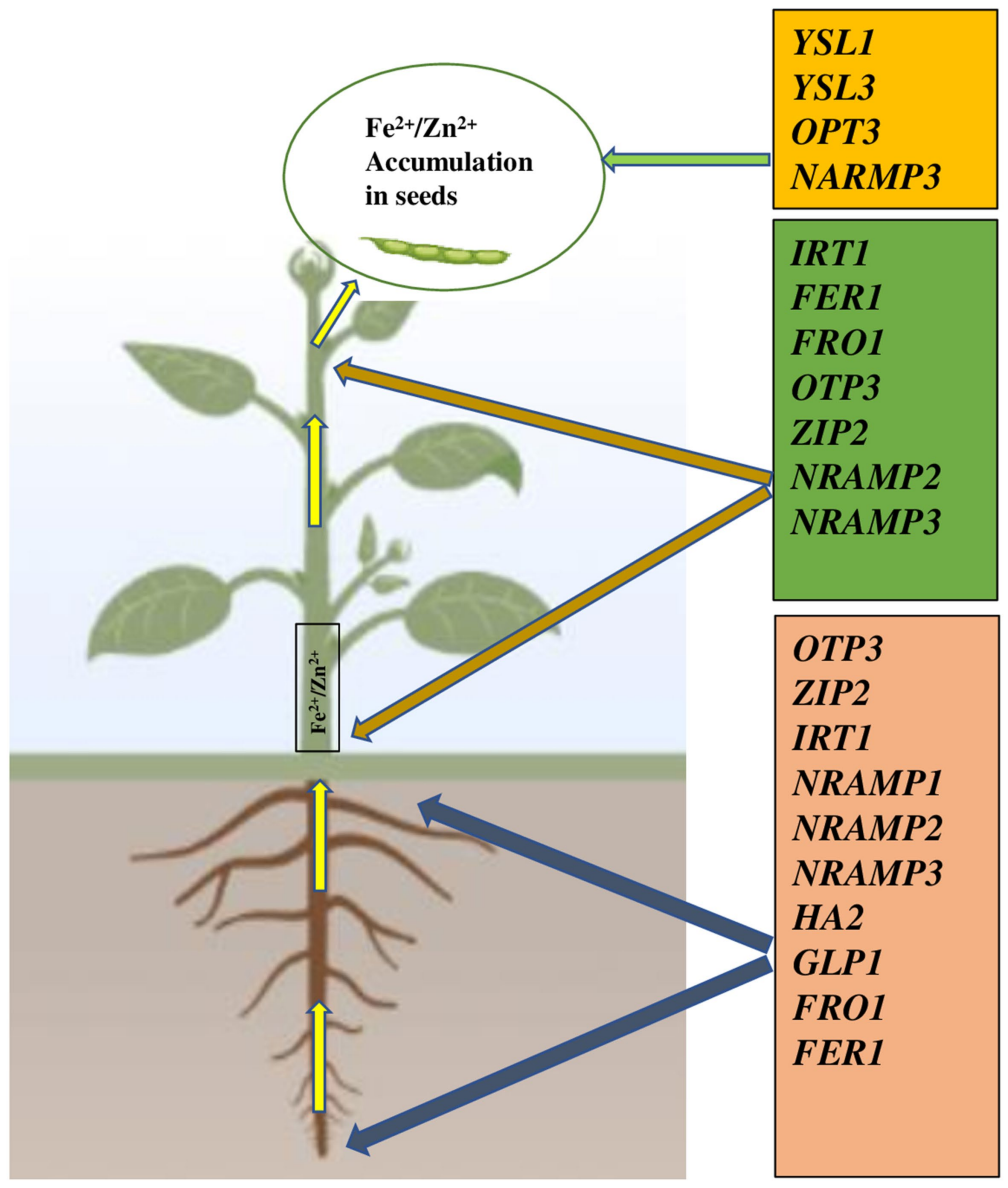
Gene families involved in translocation and accumulation of Fe^2+^/Zn^2+^ in seed of common bean (Urwat et al., 2021). *YSL1* and *YSL3* genes are involved in unloading minerals to seeds. YSL, Yellow Stripe Like; OPT, Oligopeptide transporter; NRAMP, Natural resistance-associated macrophage protein; IRT, Iron regulated transporter; FER, Ferritin; FRO Ferric reduction oxidase; ZIP, Zinc induced protein; HA, H+ transporting ATPase; GLP, Germin like protein. Plant images made with Biorender (https://biorender.com/).

More recently, in common bean, Urwat et al. (2021) demonstrated, the expression of *IRT1*, *FRO1*, and *Ferritin 1* genes showed an important role in Fe and Zn uptake/transport and signaling under Fe/Zn stress whereas the expression of *ZIP2*, *NRAMP1*, *HA2*, and *GLP1* (*Germin-like protein*) genes in *Phaseolus vulgaris* was highly responsive to Zn uptake (Figure 2).

These identified candidate gene families can be potentially deployed in crop plants to enhance the Fe/Zn uptake and its bioavailability.

## Integrated breeding and genomic approaches for biofortification of grain legumes

Conventional breeding programs require screening of a genetically diverse panel and identification of potential donor parents fortified with micronutrient(s) of our interest in order to transfer the gene to the elite genotype to obtain a biofortified crop variety (Guindon et al., 2021). Although biofortification through the use of plant breeding strategies offers the most reliable, sustainable, and cost-effective strategies, developing a bio-fortified product takes a long time. The use of molecular markers could reduce time by allowing rapid identification of the superior donor with the gene of interest.

## Genetic variation for micronutrient content in legume germplasm

Genetic diversity for micronutrient concentration in the gene pool is a prerequisite for successful biofortification. Genetic variation for micronutrient concentration has been well studied in legumes (Table 1). Research on legume crops have revealed significant genetic variations for the essential micronutrients in chickpea (Ray et al., 2014; Jha et al., 2015; Rezaei et al., 2016; Vandemark et al., 2018; Karaca et al., 2020), common bean (Beebe et al., 2000; Ray et al., 2014; Murube et al., 2021), cowpea (Gerrano et al., 2022; Sodedji et al., 2022), lentil (Ray et al., 2014; Kumar et al., 2016; Vandemark et al., 2018), mung bean (Dahiya et al., 2013; Kumar and Pandey, 2020), Pea (Ray et al., 2014; Jha et al., 2015; Dissanayaka, 2019; Klein et al., 2020) and soybean (Kumar and Pandey, 2020). The promising genotypes identified can be utilized in biofortification programs for mapping studies and development of breeding material rich in micronutrients. Molecular markers linked to micronutrient content can aid in marker-assisted selection.

**Table 1:** Genetic variability for different micronutrients in major food legumes.

Legumes	Iron (Fe)	Zinc (Zn)	Copper (Cu)	Magnesium (Mg)	Manganese (Mn)	Selenium (Se)	Boron (B)	Carotenoids	Folate	References
Chickpea	48.6–55.6 mg kg^−1^	6.6–8.7 mg kg^−1^	6.6–8.7 mg kg^−1^	1,525–1,902 mg kg^−1^	-	629–864 μg kg^−1^	-	-	-	Ray et al. (2014)
	52.75 μg g^−1^	38.91 μg g^−1^	7.42 μg g^−1^	1,402 μg g^−1^	47.47 μg g^−1^	0.35 μg g^−1^	8.29 μg g^−1^	-	-	Vandemark et al. (2018)
	60–70 mg kg^−1^	40–45 mg kg^−1^	-	-	-	-	-	-	-	Karaca et al. (2020)
	-	-	-	-	-	-	-	22–44 μg g^−1^	-	Rezaei et al. (2016)
	-	-	-	-	-	-	-	-	351–589 μg 100 g^−1^	Jha et al. (2015)
Common bean	34–89 mg kg^−1^	21–54 mg kg^−1^	14 ppm	-	29 ppm	-	-	-	-	Beebe et al. (2000)
	57.7–80.7 mg kg^−1^	24.8–33.3 mg kg^−1^	9.1–11.6 mg kg^−1^	1,845–2,383 mg kg^−1^	-	381–500 μg kg^−1^	-	-	-	Ray et al. (2014)
	55.4–176.1 mg kg^−1^	5.1–77.7 mg kg^−1^	7.0–16.5 mg kg^−1^	1.8–2.4 g kg^−1^	15.9–34.8 mg kg^−1^	0.0–4.1 mg kg^−1^	-	-	-	Murube et al. (2021)
Cowpea	83.70–109.03 and 69.77–134.16 mg kg^−1^	33.79–40.53 and 28.81 mg kg^−1^	-	-	20.60–33.83 and 18.75–36.83 mg kg^−1^	-	-	-	-	Gerrano et al. (2022)
	33.11–69.03 mg 100g^−1^	4.00–4.70 mg 100 g^−1^	-	-	14.40–19.63 mg 100 g^−1^	-	-	-	-	Gerrano et al. (2022)
								β-carotene (13.2 ± 2.9 mg 100 g^−1^) Lutein (58 ± 12.8) mg 100 g^−1^ zeaxanthin (14.7 ± 3.1 mg 100 g^−1^)		Sodedji et al. (2022)
Lentil	75.6–100 mg kg^−1^	36.7–50.6 mg kg^−1^	7.0–9.2 mg kg^−1^	938–1,071 mg kg^−1^	12.2–14.8 mg kg^−1^	-	-	-	-	Ray et al. (2014)
	42–132 ppm	23–78 ppm	-	-	-	-	-	-	216–290 μg 100 g^−1^	Kumar et al. (2016)
	73.98 μg g^−1^	50.38 μg g^−1^	9.61 μg g^−1^	10,238 μg g^−1^	17.09 μg g^−1^	0.43 μg g^−1^	-	-	-	Vandemark et al. (2018)
Mung bean	3.4–4.6 mg 100 g^−1^	1.2–2.3mg100g^−1^	-	-	-	-	-	-	-	Dahiya et al. (2013)
	3.4–4.4 mg 100 g^−1^	1.2–2.1 mg 100 g^−1^	-	129–166 mg 100 g^−1^	-	-	-	-	-	Kumar and Pandey, 2020
Pea	47.7–58.1 mg kg^−1^	27.4 34 mg kg ^−1^	5.2–6.3 mg kg^−1^	-	9.0–15.6 mg kg^−1^	405–554 μg kg^−1^	-	-		Ray et al. (2014)
	29.22–90.53 μg g^−1^	12.83–51.47 μg g^−1^	-	-	-	0.06–8.75 μg g^−1^	-	-		Dissanayaka (2019)
	22–490 mg kg^−1^	-	-	0.9–2.6 g kg^−1^	-	-	-	-	-	Klein et al. (2020)
	-	-	-	-	-	-	-	-	23–30 μg 100 g^−1^	Jha et al. (2015)

## Mapping genomic region(s)/QTL(s) for grain micronutrient content

Genetic dissection of grain micronutrient content remains complex as it is governed by multiple quantitative trait loci (QTLs). During the last decade, a paradigm shift in developing genomic resources in various legumes allowed unraveling the genetic control of important traits, including grain micronutrient content. Initially, the bi-parental mapping approach has been mainly used to investigate genomic regions controlling micronutrient content in various grain legumes (Cichy et al., 2009; Blair et al., 2010; Park et al., 2019; Diaz et al., 2020; Sab et al., 2020; Table 2).

**Table 2:** QTLs/genomics region identified/reported for various micronutrient content in legumes.

Crop	Micronutrient	Population used	QTL/genomic regions reported	Linkage group	PV explained (%)	References
Common bean						
	Zinc and Fe content	AND696 × G19833, F5:7, RIL	Single QTL for Fe and Zn	B1, B6, and B11	36%–39%	Cichy et al. (2009)
	Phosphorus content		Seed phosphorus content QTL	B1, B2, B5, B6, B8, B11	17% to 55%	
	Zinc and Fe content	G14519 × G4825, F10, RIL	Eight QTLs for Fe content, nine QTLs for zinc content	b02, b03, b04, b06, b07, b08, and b11	9.57%–55.17%	Blair et al. (2010)
	Cu, Mg, Mn, P, K, Na, S, B, and Ca	DOR364 × G19833	Four QTLs for Cu; three QTLs each for Mg, Mn, P; two QTLs each for K, Na, S, and one QTL each for B, *Ca.*	1,2,5,6,7,8,9,10, and 11	8%–29.1%	Bohra et al. (2015)
	Zinc and Fe content	AND 696 × G19833, G21242 × G21078 G14519 × G4825, Black Magic × ShinyCrow DOR 364 × G19833, BAT 93 × Jalo EEP, Cerinza × G10022	MQTL_Fe&Zn_1.1 MQTL_Fe&Zn_2.1, MQTL_Zn_2.2, MQTL_Fe&Zn_4.1, MQTL_Fe&Zn_6.1 MQTL_Fe&Zn_6.2 MQTL_Zn_7.1 MQTL_Fe_7.2 MQTL_Fe_8.1 MQTL_Fe&Zn_8.2 MQTL_Fe&Zn_9.1 MQTL_Fe&Zn_11.1	LG1, 2,4,6,9,8,11	10.3%–27.0%	Izquierdo et al. (2018)
	Fe content	MAGIC population developed from SXB412, INB827, ALB213, SEN56 SCR2, MIB778, SCR9, INB841 eight founder parents	One major QTL for Fe content	–	8.60%	Diaz et al. (2020)
Lentil						
	Selenium content	PI 320937 × Eston	SeQTL2.1, SeQTL5.2, SeQTL5.1, SeQTL5.3	LG2 and LG5	6.3%–16.9%	Ates et al. (2016)
	Zinc content	GWAS (96 lines)	Four MTAs	–	14%–21%	Singh et al. (2017)
	Iron content		Three MTAs	–	9%–11%	
	Zinc content and iron content	GWAS (138)	Two significant MTA for iron and two MTA for zinc content	–	9%–21%	Khazaei et al. (2017)
	Manganese content	RIL (CDC “Redberry” × “ILL7502”)	MnQTL1.1, MnQTL1.2, MnQTL3.1, MnQTL3.2, MnQTL3.3, MnQTL7.1	LG1, LG3, LG7	15.3%–24.1%	Ates et al. (2018)
	Zinc content	GWAS(96)	Two significant MTA	–	6%–17%	Kumar et al. (2019)
	Iron content		Three significant MTA	–	6%–13%	
Chickpea						
	Zinc content and iron content	GWAS (94 accessions)	Eight significant MTAs for grain Zn and Fe content	LG1, 4, and 7	-	Diapari et al. (2014)
	Zinc content and iron content	GWAS (92 accessions)	Eight QTLs for Fe and Zn content	LG1, LG2, LG3, LG4, LG5, and LG7	16.9–23.7	Upadhyaya et al. (2016)
	Grain iron content	MNK-1 × Annigeri1,F2:3	11 QTLs for grain Fe content	CaLG03, CaLG04, and CaLG05,	7.2–13.4	Sab et al. (2020)
	Grain zinc content		Eight QTLs for grain Zn content	CaLG04, CaLG05, and CaLG08	5.7–13.7	
Mung bean						
	Iron content	GWAS in 95 genotypes	Five significant MTA for seed Fe content	–	13.40%	Wu et al. (2020)
	Manganese content		Five significant MTA for seed Mn content	–	38.7	
	Phosphorus content		Eight significant MTA for seed P content	–	27.9	
	Sulfur content		Nine significant MTA for seed S content	–	19	
	Zinc content		Seven significant MTA for seed Zn content	–	21.8	
	Potassium content		Nine significant MTA for seed K content	–	30.4	
Pea						
	Boron content	“Aragorn” × “Kilfica” RILs	Five QTLs	LG1, 5, 6, and 7	4.3%–42%	Ma et al. (2017)
	Calcium content		Five QTLs	LG4, 5, and 7	2.4%–31%	
	Iron content		Five QTLs	LG2, 5, and 7	6.6%–19.4%	
	Magnesium content		Four QTLs	LG3, 4, and 5	4.7%–43.3%	
	Manganese content		Five QTLs	LG1, 2, 4, 5, and 7	3.6%–29.9%	
	Molybdenum content		One QTL	LG5	34.20%	
	Phosphorus content		Five QTLs	LG3, 5, and 7	5.9%–16.9%	
	Sulphur content		Five QTLs	LG3, 5, 6, and 7	5.6%–16.3%	
	Zinc content		Five QTLs	LG2, 3, 5, and 7	5.6%–12.7%	
Soybean						
	Iron content	Anoka × A7, F2:3	One major QTL for Fe	LG20	21.50%	King et al. (2013)
	Tocopherol content	Tocopherols contentTK780 × B04009	Six QTLs	LG9, 11, and 12	up to 56.4%	Park et al. (2019)

Availability of high throughput novel single-nucleotide polymorphism (SNP) markers allows implementations of GWAS approach for identifying genomics regions/haplotype assembly rich in micronutrient density in various grain legumes (Diapari et al., 2014; Khazaei et al., 2017; Diaz et al., 2020; Wu et al., 2020). Employing SNP markers derived from genotyping by sequencing technique in a chickpea mapping population derived from the cross MNK1 x Annigeri1 a total of eight QTL contributing to grain Fe content on CaLG03, CaLG04, and CaLG05 and a total of 11 QTLs controlling grain Zn content on CaLG04, CaLG05, and CaLG08 were identified (Sab et al., 2020). Notably, they also found three QTLs *viz.*, CaqFe4.4, CaqFe4.5, and CaqZn4.1 were colocalized on the “QTL-hotspot” region for drought tolerance on CaLG04 (Sab et al., 2020). In a study on a diverse set of chickpea genotypes, GWAS analysis recruiting 1,536 SNPs markers revealed one significant MTA for grain Zn on LG1, three significant MTAs for grain Zn on LG4, two important MTA for Fe content on LG4, and two significant MTA for grain Fe and grain Zn content on LG6 and LG7 (Diapari et al., 2014).

Phenotypic and genotypic evaluation of a mapping population developed from the cross AND969 x G19833 in common bean revealed a significant QTL controlling grain Zn and Fe content colocalized on B6 chromosome and contributing 36% of total phenotypic variation (Cichy et al., 2009). Subsequently, a total of 12 meta-QTLs controlling grain Zn and Fe content were identified by assessing seven mapping populations tested across multi-locations. Among these meta-QTLs, 2 QTLs were found to be specific to grain Fe and two QTLs specific to grain Zn and eight QTLs remained colocalized controlling both Fe and Zn content (Izquierdo et al., 2018). QTL analysis in a multiparent advanced generation intercross (MAGIC) population derived from eight founder parents allowed pinpointing two QTLs (*SdFe6.1* and*SdFe6.2*) controlling grain Fe content in common bean (Diaz et al., 2020). Genetic dissection of micronutrients in common bean unraveled 21 QTLs in an F_9:11_ RIL (DOR364 × G19833) population for 13 nutrient elements (Blair et al., 2016). In common bean, a genome-wide survey through SNP markers in a set of 192 accessions resulted in one significant MTA for grain Zn on the PV01 chromosome (Caproni et al., 2020). Further, the underlying candidate gene controlling grain Zn content was *Phvul001G233500.*

In lentils, a total of six QTLs controlling grain Mn content explaining phenotypic variation up to 24% were elucidated on LG1, LG3, and LG7 by evaluating mapping population derived from CDC Redberry × ILL7502 cross assessed for 2 years at three locations in Turkey (Ates et al., 2018). Mapping for Se content in lentil recombinant inbred lines (RILs) developed from PI 320937 × Eston cross identified a total of 4 QTLs on LG2 and LG5 explaining 6.3%–16.9% phenotypic variation (Ates et al., 2016). In a marker-trait-association (MTA) study in lentil, mapping in a panel comprising 96 diverse germplasm for Fe and Zn concentration identified three markers PBALC 13, PBALC 206, and GLLC 563 associated with grain Fe concentration that explained 9%–11% of phenotypic variation and four SSR markers PBALC 353, SSR 317–1, PLC 62, and PBALC 217 associated with grain Zn concentration which explained 14%–21% of phenotypic variation (Singh et al., 2017). Subsequently, association mapping for grain zinc and iron content by deploying 80 SSR markers in a panel of 96 lentil genotypes allowed deciphering two significant marker-trait associations for grain Fe and three significant marker-trait associations for grain Zn content (Kumar et al., 2019). Two significant MTAs for grain Fe content and one important MTA for grain Zn were identified through deploying 1,150 SNP markers in a panel of 138 lentil accession collected from diverse countries (Khazaei et al., 2017). GWAS in a set of 1,150 individuals derived from MAGIC population, evaluated in 2014 and 2016 revealed two QTLs (*SdFe6.1*, *SdFe6.2*) controlling grain Fe content (Diaz et al., 2020).

In a GWAS study performed in a set of 96 mung bean genotypes collected from 13 countries five significant MTAs for Fe content, five for Mn content, eight for P content, nine for S content, seven for Zn content, and nine for K content were identified.

## Exploitation of miRNAs for micronutrient enhancement

MicroRNAs (miRNAs) are potential candidates for increasing grain micronutrients, in addition to protein-coding genes (Sunkar and Zhu, 2004). miRNAs are involved in almost every biological and metabolic process in plants, including development, cell wall production, and stress responses (Chen, 2009). miRNA are one of the potential tools to enrich grain particles with micronutrients by regulating gene expression in food crops. To date, several miRNAs have been discovered with regulatory activity in differentially expressed genes that governs various functions such as uptake, mobilization, and homeostasis of nutrients, ensuring adequate supply without any toxicity and also reported their specific roles in homeostasis of micronutrients like B, Mn, Zn, Cu, Mo, and Ni (Patel et al., 2017).

miRNAs have emerged as important regulators of plant stress responses and for maintaining nutrient homeostasis by regulating the expression of transporters involved in nutrient absorption and mobilization (Paul et al., 2015). miRNAs also have a role in nutritional homeostasis by acting as an endogenous signal for micro and macronutrient availability (Marín-González and Suárez-López, 2012; Kehr, 2013; Jagadhesan et al., 2022). The reports on miRNAs regulating micronutrient homeostasis in legumes are meager, barring the information pertaining to *Phaseolus vulgaris* (common bean) and *Medicago truncatula* (Valdes-Lopez et al., 2010; Naya et al., 2014).

During Copper (Cu) deficiency, several miRNA families, such as miR397, miR408, and miR857 are upregulated, suppressing the production of laccase and plastocyanin genes (Abdel-Ghany and Pilon, 2008). The induction of miR398 suppresses Cu/Zn superoxide dismutase CSD1, CSD2, and Cu chaperones for SOD1 (CCS1) gene expression under Cu deficit (Sunkar et al., 2006). When exposed to Cu deficiency, common bean plants with transgenic roots over-expressing miR398 showed a 20-fold increase in mature miR398b and almost no target transcript levels, as well as increased anthocyanin content and expression of Cu-stress responsive genes (Naya et al., 2014). Fe deficit also regulates miR398, one of the primary activators of CSD gene expression during Cu deficiency, albeit in the opposite direction. Cu shortage increases the expression of miR397, miR398a, miR398b, miR398c, miR398s, miR399, miR408, and miR2111, whereas Fe deficiency decreases their expression and hence regulates CSD1 and CSD2 expression (Pant et al., 2008; Waters et al., 2012). As a result, the Cu-Fe interaction is yet another novel finding in the field of Fe homeostasis gene expression research. In addition, eight miRNAs from five families (miR159, miR164, miR172, miR173, and miR394) were previously identified as Fe-responsive families in Arabidopsis, The Fe deficiency responsive cis-acting elements1 and 2 (IDE1/IDE2) were discovered in the promoters of 24 miRNA genes in Arabidopsis, and they mirror the Fe-responsive gene families that are controlled during Fe deficiency (Kong and Yang, 2010).

Valdes-Lopez et al. (2010) found miRNAs in the leaves, roots, and nodules of control and nutrient-stressed common bean plants (deficiency of phosphorus (P), nitrogen (N), or iron (Fe); acidic pH; and manganese (Mn) toxicity). Cu content was shown to be higher in common bean plants grown under N and Fe deficit conditions, where miR398 and miR408 were downregulated, but lower in acidic pH and high Mn, where miR398 was upregulated. These findings are consistent with those of Yamasaki et al. (2009), who discovered that SQUAMOSA promoter binding protein-like 7 (SPL7) increased the transcription of miR397, miR398, and miR408 in *Arabidopsis* under Low-Cu circumstances. For example, pvu-miR1511, gma-miR1513, gma-miR1515, and gma-miRNA1516 were all found to be significantly enhanced in Fe deficient leaves, suggesting that they may be involved in the Fe signal transduction pathway (Valdes-Lopez et al., 2010). Several miRNAs have been discovered to be upregulated during Mn deficiency which are linked to other mineral deficiencies also. miR319, miR169, miR396, miR170, miR164, miR390, miR395, miR166, miR172, miR157, miR156, and miR167 are upregulated during Mn toxicity in *Phaseolus vulgaris* and suppresses the expression of a wide group of genes, including various transcription factors such as TEOSINTE-LIKE1, CYCLOIDEA, PROLIFERATING CELL FACTOR1 (TCP), HAPLESS (HAP2), SCARECROW-LIKE, NO APICAL MERISTEM (NAC; Valdes-Lopez et al., 2010).

High concentration of Al, Cd, Hg, and Cd, have also been shown to cause upregulation or downregulation of certain miRNAs, which are thought to play a role in plant adaptation processes by regulating the expression of numerous stress-related genes (Zhou et al., 2012; Li et al., 2013; Zeng et al., 2014). In Arabidopsis sulfate, P, and Cu deprivation stimulate miR395, miR399, and miR398 expression (Sunkar et al., 2007). In Arabidopsis and Brassica, Cu deficiency has been found to enhance the production of miR397 and miR408 genes (Sunkar et al., 2006; Naya et al., 2014). When three-week-old *Medicago truncatula* seedlings were subjected to deficiency of sulfate, P, or Cu. In response to sulfate deficiency, the abundance of miR395 was increased in shoots and roots. When grown on phosphate-free media, miR399 was activated, while miR398 was upregulated in both shoots and roots of *M. truncatula* grown on medium without Cu. A similar pattern of induction was seen for miR397 in *M. truncatula* seedlings grown on Cu-deficient media (Jagadeeswaran et al., 2009; Saini et al., 2012). Mineral ion absorption, transport, and remobilization are all closely related to mineral bioavailability in grains (Aung et al., 2013). The discoveries on nutrient-related miRNAs and their gene regulation mechanism could pave the way for a new platform for boosting legume bio fortification initiatives in the future (Blair et al., 2013).

## Biofortification through agronomic interventions

Agronomic intervention optimizes the application of mineral fertilizers and improves the solubilization and mobilization of mineral elements in the soil (White and Broadley, 2009). Application of fertilizers can improve the micronutrient status in soil to overcome micronutrient deficiencies in humans (Cakmak, 2008). The use of mineral fertilizers is feasible in the developed world, as demonstrated by the success of selenium (Se) fertilization of plants in Finland (Aro et al., 1995), zinc (Zn) fertilization in Turkey (Cakmak et al., 1999) and iodine (I) fertilization in irrigation water in China (Jiang et al., 1997). Biofortification with agronomic approaches has been successfully demonstrated in some legumes. For example; the application of Se as selenate provides more phyto-availability than selenite in cowpea (Silva et al., 2019). A recent study found that combined foliar treatment of ZnSO_4_.H_2_O (0.5%) and FeSO_4_H_2_O (0.5%) at the pre-flowering and pod formation stages in lentil were most effective in enhancing grain and straw yield, Zn and Fe content, and uptake (Dhaliwal et al., 2021). Likewise, the foliar application of a mixture containing 8 mg ZnSO_4_·7H_2_O/kg soil and 0.25% (w/v) ZnSO_4_·7H_2_O could serve a better option for fortifying field peas (Poblaciones and Rengel, 2016). Similarly, Zn-fortified soybean sprouts have been successfully achieved by application of ZnSO_4_ solution, containing, Zn 10 to 20 μg/ml (Zou et al., 2014).

Use of agronomic practices like intercropping, accelerate the P, Fe and Zn uptake efficiency, leading to increased yield and improved grain quality (Xue et al., 2015). Biofortification through agronomic approaches is simple and the least expensive but has some disadvantages like, regular application of mineral fertilizers, could impede the accessibility of other micronutrients and also cause environmental toxicity (Jha and Warkentin, 2020). In addition, soil factors such as higher pH, calcite, organic matter, and high concentration of other ions hinder the accessibility of micronutrients to the crop plants (Alloway, 2009).

## Transgenic and genome editing technologies for biofortification

In crops, in which the desired variation for a nutrient is not available in cultivated and wild relatives or in which biofortified crop varieties are difficult to produce with the help of conventional breeding approaches, transgenic breeding and genome editing are alternative approaches to produce bio-enriched crops with nutritional traits of interest. In addition to enriching the micronutrient concentration, this approach can also target the removal of Anti-nutrients or the ingestion of promoters that can increase the bioavailability of micronutrients (Carvalho and Vasconcelos, 2013; Garg et al., 2018). Genome editing is a powerful tool for knock out, knock down, or alteration of gene expression without disturbing the genetic makeup of the genotype. CRISPR/Cas9 genome editing can be utilized to enhance the seed micronutrient content as well as to reduce anti-nutritional factors in plants.

CRISPR-edited plants are free from any foreign DNA; thus, they may have better acceptability compared to traditional GM crops. Targeted genome editing through the use of artificial nucleases such as zinc-finger nucleases (ZFNs), transcription activator-like effector nucleases (TALENs) has the potential to accelerate basic as well as applied research by offering the possibility to quickly and precisely create modified genomes in a predictable way (Bortesi and Fischer, 2014; Jaganathan et al., 2018). Unlike first-generation genome editing tools (ZFNs and TALENs), clustered regularly interspaced short palindromic repeat (CRISPR)/CRISPR associated protein Cas9 genome editing represents simple designing and cloning techniques, Cas9 protein with guide RNAs potentially targeting multiple sites in the genome (Jaganathan et al., 2018). Applying these modern genome editing techniques will aid in the development of non-GMO plants with the desired trait that can contribute to increased yield potential under stressful conditions and also improve the nutritional quality. In recent years, genome editing techniques have been applied on several crops like rice (Li et al., 2012; Zhang et al., 2014) wheat (Wang et al., 2014), and tomatoes (Brooks et al., 2014). In addition, transgenic rice with 30% of the estimated average requirement (EAR) for micronutrients Fe and Zn has been developed and tested in flooded field conditions (Trijatmiko et al., 2016). In rice, for Fe and Zn biofortification, the nutritional goals were achieved in field conditions in the Philippines and Colombia (Trijatmiko et al., 2016). Transgenic corn containing multivitamins was made by modifying three different metabolic pathways at the same time to increase levels of three vitamins, namely beta-carotene (169-fold), ascorbate (6-fold), and folate (2-fold), in the endosperm (Naqvi et al., 2009). Metabolic engineering has increased the folate concentration in tomatoes and rice (Blancquaert et al., 2013, 2014). A recent study demonstrated the synergistic use of CRISPR/Cas9 and TALENs technologies to generate a collection of functional mutant plant lines in *Glycine max* and *Medicago truncatula* (Curtin et al., 2018). In wheat, the CRISPR/Cas9 editing of TaIPK1 effectively reduced the phytate content and thereby increased the grain Fe and Zinc (Zn) content (Ibrahim et al., 2022). Nucleotide substitutions of inositol trisphosphate six kinase gene (*OsITPK6*) could significantly reduce rice grain phytic acid content. Targeted editing of the first exon of *OsITPK6* using the CRISPR/Cas9 method generated mutant lines *OsIPTK6*_1 and *OsIPTK*6_2 with significantly low phytic acid content and higher inorganic P levels than the wild type (Jiang et al., 2019).

## Recent success in legume biofortification with genome editing

The success in developing transgenic fortified pulses has not yet been reported, due to the absence of effective and reproducible plant regeneration methods and biosafety regulations. Recently, genetically engineered chickpea with high Fe content using chickpea *nicotianamine synthase 2* (*CaNAS2*) gene in combination to soybean *ferritin* (*GmFER*) genes was developed (Tan et al., 2018). Transgenic chickpea lines with root-specific expression of the *cytokinin oxidase/dehydrogenase 6* (*CaCKX6*) gene had increased concentration of minerals, namely Zn (27%–62%), Cu (26%–61%), Fe (22%–48%), Mg (13%–22%), K (11%–27%), and P (5%–19%) (Khandal et al., 2020). The NAS genes isolated from chickpea (*CaNas2*) and rice (*OsNas2*) catalyze the biosynthesis of nicotianamine (NA) and are involved in Fe uptake and translocation in plants. Stable transgenic chickpea lines containing GUS (*uidA*) as well as Fe-biofortification genes (*OsNAS2* and *CaNAS2*) have successfully been produced (Das Bhowmik et al., 2019). Transgenic common bean expressing *methionine-rich storage albumin* gene from Brazil nut showed increased methionine and cysteine content (Aragao et al., 1999). Enhanced expression of sunflower seed albumin gene increased methionine content (by up to 94%) in lupin (Molvig et al., 1997).

In cowpea (*V. unguiculata*), symbiotic nitrogen fixation was successfully disrupted by targeting a symbiosis receptor-like kinase gene using CRISPR/Cas9-mediated gene editing (Ji et al., 2019). In soybean, instance of increasing pro-vitamin A, oleic acid, and seed protein content in soybean by expressing bacterial *PSY* (phytoene synthase) gene is available (Schmidt et al., 2015). Likewise, improvement of beta-carotene through overexpression of *PSY* and carotene desaturase in soybean has been reported (Kim et al., 2012). Soybean protein is deficient in sulfur-containing essential amino acids *viz.*, cysteine, and methionine. Kim et al. (2012) demonstrated increased cysteine content through over expression of *O-acetylserine sulfhydrylase* gene in soybean. Overexpression of maize zein protein enabled in enhancing methionine and cysteine content in soybean (Dinkins et al., 2001). Enhanced methionine content in soybean through overexpression of cystathionine γ-synthase has been reported (Hanafy et al., 2013; Song et al., 2013). Manipulation of maize C1 and R transcription factor-driven gene enhanced isoflavone content in soybean (Yu et al., 2003).

The development of RNA-guided genome editing (CRISPR-Cas9 technology) has been established in some legume crops, like *Medicago truncatula*, soybean, cowpea, and chickpea for which transformation protocols are available. Multiple GmU6 promoters were tested for their suitability in driving gRNA expression in soybean hairy roots (Di et al., 2019). It was found that the GmU6-8 and GmU6-10 promoters had higher activity in enhancing editing efficiency (20.3% and 20.6%, respectively), compared with the other nine promoters. Gene editing has been successfully carried out by CRISPR/Cas9 technology in soybean for seed-related traits, such as seed oil profile (Do et al., 2019), unpleasant beany flavor of seed product (Wang et al., 2020), and isoflavone content and resistance to soybean mosaic virus (Zhang et al., 2020).

Gene-editing through CRISPR/Cas9 technology has paved way for novel possibilities for functional genomics in grain legume crops. The basic requirement for genetic transformation, including CRISPR/Cas9 gene editing, is the ability to deliver the DNA/RNA components, with the regeneration of an entire plant. Most of the food legumes are resistant to the uptake and integration of introduced DNA (Yadav et al., 2017) and quite a few of them are also recalcitrant (Ochatt et al., 2018). Hence, the availability of effective methods for plant transformation and whole-plant regeneration along with a supportive regulatory environment, and consumer acceptance of gene-edited crops are necessary for the success of genome editing for legume enhancement.

## Leads in pulses biofortification

Biofortification strategies have contributed to food security by producing legume varieties fortified with essential micronutrients (Table 3).

**Table 3:** Varieties/technologies in legumes through different biofortification approaches.

Legumes	Fortified elements	Status and country of release	Product/varieties	References
**Agronomic intervention**				
Chickpea				
	Fe, Ca, Cu, Mn, and Mg	Research	–	Sathya et al. (2016)
	Fe and Zn	Research	–	Pellegrino and Bedini (2014)
	Se	Research	–	Poblaciones et al. (2014)
	Zn	Research	–	Shivay et al. (2015)
Common bean				
	N, P, K, Cu, Mn, and Zn	Research	–	Westermann et al. (2011)
	Zn	Research	–	Ibrahim and Ramadan (2015); Ram et al. (2016)
Cowpea				
	Se	Research	–	Ramos et al. (2019)
Pea				
	Zn	Research	–	Poblaciones and Rengel (2016)
Soybean				
	Se	Research	–	Yang et al. (2003)
**Breeding**				
Beans				
	Fe	Released, Rwanda	RWR 2245, RWR 2154, MAC 42, MAC 44, CAB 2, RWV 1129, RWV 3006, RWV 3316, RWV 3317, and RWV 2887	HarvestPlus, Rwanda (https://www.harvestplus.org/rwandasuccess)
	Fe	Released, Democratic Republic of Congo	COD MLB 001, COD MLB 032, HM 21–7, RWR 2245, PVA 1438, COD MLV 059, VCB 81013,Nain de Kyondo, Cuarentino, Namulenga	HarvestPlus, Democratic Republic of Congo
Cowpea				
	Fe	Released, India	Pant Lobia-1, Pant Lobia-2, Pant Lobia-3, and Pant Lobia-4	HarvestPlus, GB Pantnagar University, India
Lentil				
	Fe and Zn	Released, Bangladesh	Barimasur-4, Barimasur-5, Barimasur-6, Barimasur-7, Barimasur-8, and Barimasur-9	HarvestPlus, ICARDA (https://www.icarda.org/media/blog/lentil-biofortification-research-fight-hidden-hunger)
		Released, Nepal	ILL 7723, Khajurah-1, Khajurah-2, Shital, Sisir Shekhar, Simal and Khajurah Masuro-4	
		Released, India	L4704, Pusa Vaibhav	
		Released, Ethiopia	Alemaya	
		Released, Syria	Idlib-2, Idlib-3	
Transgenic				
Chickpea				
	Fe	Research	–	Tan et al. (2018)
	Fe, Zn, Cu, Mg, K, and P	Research	–	Khandal et al. (2020)
**Commonbean**				
	Methionine	Research	–	Aragao et al. (1999)
**Lupins**				
	Methionine	Research	–	Molvig et al. (1997)
**Soybean**				
	beta-carotene	Research	–	Kim et al. (2012); Schmidt et al. (2015)
	Cysteine	Research	–	Kim et al. (2012)
	Flavonoids	Research	–	Yu et al. (2003)
	Methionine and cysteine Methionine	Research	–	Dinkins et al. (2001); Hanafy et al. (2013); Song et al. (2013)
	STA	Research	–	Eckert et al. (2006)

Biofortified varieties of lentils have been developed as part of the CGIAR’s Harvest Plus program. Nepal has released Khajurah Masuro-4 with an Fe content of more than 75 ppm.^1^ Lentil varieties enriched with Fe and Zn content from Bangladesh are Barimasur-9 (78 ppm Fe), Barimasur-4 (86.2 ppm, Fe), Barimasur-5 (86 ppm Fe and 59 ppm Zn), Barimasur-6 (86 ppm Fe and 63 ppm Zn), Barimasur-7 (81 ppm Fe) and Barimasur-8 (75 ppm Fe and 60 ppm Zn). In India biofortified lentil varieties L4704 (125 ppm and 74 ppm) and Pusa Vaibhav (102 ppm Fe) have been released for commercial cultivation while IPL 220 (113 ppm Fe) has been recently identified. Idlib-2 (73 ppm Fe) and Idlib-3 (72 ppm Fe) from Syria while Alemaya (82 ppm Fe and 66 ppm Zn) have been released from Ethiopia.

Cowpea enriched with Fe (Pant Lobia-1, Pant Lobia-2, Pant Lobia-3, and Pant Lobia-4) has been developed from G.B. Pant University of Agriculture and Technology, Pantnagar (India).

Similarly, agronomic practices have supported some important achievements in legumes (Table 3). In chickpeas, Fe and Zn have been fortified by use of arbuscular mycorrhizal fungi (AMF; Pellegrino and Bedini, 2014). Biofortification of chickpeas with Zn and Se by foliar application has also been reported (Poblaciones et al., 2014; Shivay et al., 2015). Zinc biofortification by foliar application of Zn fertilizer has been described in common bean (Ibrahim and Ramadan, 2015; Ram et al., 2016). Soybean fortified with Se was obtained by foliar application of fertilizer complexed with selenium (Yang et al., 2003). Recently, foliar application of Na_2_SeO_4_ as a source of Se in cowpea for biofortification has been reported (Ramos et al., 2019).

## Anti-nutritional factors in legumes

## Phytic acid

Phytic acid (PA) is one of the most important anti-micronutrient component in grain which reduces the bioavailability of micronutrients. Ingestion of PA-rich foods leads to deficiencies of minerals such as Fe and Zn in the human population (Pramitha et al., 2021). In legume seeds, phytic acid is commonly present as myoinositol 1,2,3,4,S,6-hexakis (dihydrogen phosphate; Urbano et al., 2000). Phytate (IP6) is a compound that comprising of six phosphate groups connected to an inositol ring that can bind up to 12 protons. The Anti-nutritional effect is attributed to these six phosphate groups which are powerful chelators, binding rapidly to mineral cations mostly Cu^2+^, Ca^2+^, Zn^2+^, and Fe^3+^ (Castro-Alba et al., 2019). Phytic acid limits the utilization of phosphate as it is present mainly in the organic form of inositol hexakisphosphate (IP6). Hence, low IP6 content can improve crops and grains’ phosphate and mineral bioavailability. Majority of studies on impact of phytate on mineral nutrition suggest that insoluble phytate-metal complexes are formed in the digestive track, preventing metal absorption. The reduced availability of essential minerals in legumes and other protein foods depends on several factors, such as the ability of endogenous carriers in the intestinal mucosa to absorb the essential minerals bound to phytate and other dietary substances, phytic acid, mineral concentrations in foods, phytate hydrolysis by the phytase enzyme in the intestine, phytate inhibition, and food processing methods (Rackis and Anderson, 1977).

Variation in phytic acid content has been detected in legumes. The concentration of phytic acid in legumes grown in Canadian environment ranged from 11.33–14 mg/g in chickpea,15.64–18.82 mg/g in common beans,19.65–22.85 mg/g in faba bean, 8.56–17.1 mg/g in lentil and 9.93–12.40 mg/g in peas (Shi et al., 2018). Recent study demonstrated that cotyledon of common bean is rich in minerals Mg, Ca, Fe, and Zn but it also contain a significant amount of phytic acid that limits mineral absorption during digestion (Rousseau et al., 2020). Chickpea and pigeon pea have low phytic acid contents. Breeding programs should use low-phytic acid genotypes of pulses in order to increase their nutritional value and uptake (Chitra et al., 1995). Significant role of polyamines have been observed in breakdown of phytate. Polyamine spermidine (Spd), could hasten phytic acid breakdown in mung bean sprouts (Zhou et al., 2017). In faba bean, it was observed that phytic acid create very persistent compounds with proteins and making them inaccessible to gastrointestinal digestion and absorption (Rosa-Sibakov et al., 2018). Therefore phytase treatment may be advantageous in plant-based foods where high phytic acid levels may prevent proper protein digestion. Processing techniques such as soaking, fermentation, sprouting, germinating, and cooking procedures can drastically change the phytate content of grains in legumes, allowing improved mineral bioavailability (Petroski and Minich, 2020). For example, cooking legumes for 1 h at 95°C resulted in phytate reduction up to 23% in yellow split peas, 20%–80% in lentils, and 11% in chickpeas. In black beans, just a 0.29% reduction was observed (Shi et al., 2018). In legumes, breeding strategies have been attempted to reduce the PA content. For example, in common beans, low phytic acid (*lpa*) mutant lines were derived from a mutagenized population with up to 90% reduced PA levels (Campion et al., 2009). A low phytic acid line (*lpa1*) of common bean has been identified and used to map the *Mrp1* gene, which is involved in the metabolism of phytic acid (Panzeri et al., 2011). Similarly, low-phytate pea (*Pisum sativum* L.) lines have been developed using chemical mutagenesis in cultivar CDC Bronco (Warkentin et al., 2012).

### Raffinose family oligosaccharides

Raffinose series oligosaccharides (RFO’s) are another major anti-nutrients in legumes that shows significant Anti-nutritional effects in monogastric animals and human beings. In contrast to phytic acid which chelates minerals and decreases their bioavailability to humans and other animals, RFOs are responsible for flatulence (Zhawar et al., 2011). In chickpea, considerable amount of raffinose sugars were found (0.38–0.99 g/100 g; Roorkiwal et al., 2021). Efforts are being made to reduce phytic acid and RFO content without compromising on other quality parameters in chickpea.

### Oxalates

Oxalates can form insoluble salts with sodium, potassium, calcium, iron, and magnesium. Both water soluble and insoluble oxalates are derived from plant. Water-soluble oxalate are potassium oxalate, sodium oxalate, and ammonium oxalates while insoluble oxalate is calcium oxalate (Noonan and Savage, 1999). According to Savage et al. (2000), oxalates that are soluble can chelate minerals and reduce absorption, or can lead to formation of calcium oxalate kidney stones. Due to negative effect in mineral absorption and health ailments oxalates are considered as Anti-nutritional factors. According to Shi et al. (2018), soybeans had the highest concentration of oxalates (370 mg/100 g dry weight (DW)), followed by lentils and peas (168–293 mg/100 g DW), chickpeas (192 mg/100 g DW), and common beans (98–117 mg/100 g DW). However, studies have shown that cooking can effectively reduce the amount of soluble oxalate that is present in the diet (Petroski and Minich, 2020).

### Tannins

Tannins, in the form of proanthocyanidins or catechins are considered one of the most prevalent secondary plant metabolites that are found in cocoa beans, tea, wines, fruits, juices, nuts, seeds, legumes, and cereal grains (Petroski and Minich, 2020). Tannins serve as antioxidants by scavenging free radicals, and has also ability to chelate dietary elements like Fe^2+^,Cu^2+^, and Zn^2+^ (Karamac, 2009). It has been demonstrated that kidney bean (*Phaseolus vulgaris* L.) contained a significant amount of catechins (20.1 mg/kg; Arts et al., 2000). Processing and cooking could reduce the total catechin content in food crops.

### Saponins

Saponins are a group of amphipathic compounds composed of lipophilic triterpene and hydrophilic sugar moieties. Pea seeds biosynthesize saponin B (also referred to as soyasaponin I) and DDMP saponin (soyasaponin βg; Reim and Rohn, 2015). The presence of saponin B and DDMP saponin in pea flour causes astringent, and bitter “off-flavor” (Heng et al., 2006). As the biosynthetic genes for these compounds have been reported in legumes (Sundaramoorthy et al., 2019), CRISPR/Cas9 can be applied to either remove or reduce the levels of saponin B and DDMP saponin in pea seeds.

## Conclusion and future perspective

Evaluation of genetic resources in legume crops has revealed significant variation for protein content as well as micronutrients. This review highlights the efforts being made toward screening of germplasm, mapping of QTLs for seed protein and micronutrient content and future thrust areas for enhancing the nutritional quality of major pulses. Micronutrient content in seed appears to be a complex trait under control of number of genes/QTLs and the concentration of these nutrients is highly influenced by multiple factors which include edaphic as well as environmental components. An integrated breeding approach involving QTL mapping, genome-wide association analysis, and differential gene expression profiling is currently the most efficient strategy for genetic dissection of complex traits like nutritional quality.

Ample scope exists for further improving the nutritional quality of pulse crops and development of biofortified varieties. The variability for Fe and Zn content and other micronutrients in food legumes can be exploited for enhancing the micronutrient level and their bioavailability. Minimizing the anti-nutritional factors *viz.*, phytate, trypsin, and chymotrypsin inhibitors can further enhance the bioavailability and consumption of legumes. Search for genotypes with low anti-nutrient content in the germplasm or through induced mutagenesis or genome editing is an important objective in legume improvement program for achieving nutritional security. Crop wild relatives of various legumes could be used in pre-breeding program as they lack these Anti-nutrients (Singh and Jauhar, 2005).

The whole genome information coupled with phenotypic evaluation can identify rare variants for nutritional quality traits. The identification of haplotypes governing high protein and micronutrient content, low anti-nutritional factor in legume crops will be useful for translation genomics to breed biofortified cultivars with superior nutritional quality. Recent findings represent the kudos for future application of transgenic and gene editing approaches in legumes for enhancement of nutritional quality traits.

## Author contributions

RJ and ST conceived the idea. RJ, UJ, HY, and RR prepared the manuscript. LS, PS, RS, AS, MT, UJ, SC, and ST edited and finalized the manuscript. All authors contributed to the article and approved the submitted version.

## Conflict of interest

The authors declare that the research was conducted in the absence of any commercial or financial relationships that could be construed as a potential conflict of interest.

The reviewer KT declared a shared affiliation with the authors ST, RJ, HY, RR, RS, UJ, and LS at the time of the review.

## Publisher’s note

All claims expressed in this article are solely those of the authors and do not necessarily represent those of their affiliated organizations, or those of the publisher, the editors and the reviewers. Any product that may be evaluated in this article, or claim that may be made by its manufacturer, is not guaranteed or endorsed by the publisher.

## Footnotes

^1^ https://www.icarda.org/media/blog/lentil-biofortification-research-fight-hidden-hunger
